# Contributions to the Therapeusis of Cholelithiasis

**Published:** 1908-02

**Authors:** 


### Contributions to the Therapeusis of Cholelithiasis

ARTHUR SCHWEITZER (*Port and School Physician,* Fiume; *Gyogyaszat,* Budapest, March 17, 1907), says a survey of current medical literature leads to the gratifying conclusion that important progress has been made both in regard to the etiology and treatment of cholelithiases. It is now established that it is due to catarrh of the gall-bladder and ducts, occasioning deposits of lime and cholesterin. Biliary stasis hinders expulsion of concretions and of microörganic accumulations; the disease occurs in 20.6 per cent. of females who wear corsets, and only in 4.4 per cent. of males.

Carlsbad mineral waters, as Naunyn and others have shown, act by stimulating the circulation and increasing the powers of resorption, and by favorably influencing the hepatic and gastrointestinal mucosæ. But even the warmest advocates of Carlsbad admit that radical cure can only be expected from drinking the waters right at the springs, whereas many sufferers are unable to leave home.

Salicylic acid produces an abundant flow of thin bile and has important antipastic and antiseptic effects. It has no direct solvent action, but does prevent the formation of new stones. The employment of olive oil, though often followed by excellent results, is extremely difficult on acount of the uncontrollable nausea it occasions. Blum therefore advocated the nonirritating saponaceous combination, the oleate of soda, which in addition to the cholagogue action of the oil has marked cholesterin-solvent effect.

The favorable results which Brings and others obtained from probilin pills, in which Bauermeister effected a happy combination of salicylic acid and sodium oleate, induced Schweitzer to try the remedy in his own practice in seven cases, of which he details three. The attacks diminished in number and intensity even in the first days of the probilin treatment; and after its termination the patients remained well for considerable periods of time. It is well to employ the pills also as a prophylactic measure.

Acute cases should get twenty probilin pills a day; chronic ones six to ten daily, three to five morning and evening. In chronic cases a four weeks' course, followed by a short interval, and a three weeks' course four times during the same year, are advisable. Schweitzer concludes that Bauermeister's pills are undoubtedly remarkably efficacious in all varieties of gallstone disease, and he is convinced they will soon come into general employment.

